# PCR-Based Detection of *Babesia ovis* in *Rhipicephalus bursa* and Small Ruminants

**DOI:** 10.1155/2014/294704

**Published:** 2014-04-24

**Authors:** Bijan Esmaeilnejad, Mousa Tavassoli, Siamak Asri-Rezaei, Bahram Dalir-Naghadeh, Karim Mardani, Ghader Jalilzadeh-Amin, Mostafa Golabi, Jafar Arjmand

**Affiliations:** ^1^Department of Pathobiology, Faculty of Veterinary Medicine, Urmia University, Urmia, Iran; ^2^Departments of Clinical Sciences, Faculty of Veterinary Medicine, Urmia University, Urmia, Iran; ^3^Department of Food Hygiene and Quality Control, Faculty of Veterinary Medicine, Urmia University, Urmia, Iran

## Abstract

This study aimed to assess the prevalence of *Babesia ovis* infection in adult *Rhipicephalus bursa* and small ruminants in West Azerbaijan province, Iran. Blood samples were collected from 280 sheep and 122 goats of forty randomly selected flocks. Specific *B. ovis* fragment was detected in 67 animals (16.7%), of which 52 animals (18.6%) were sheep and 15 animals (12.2%) goats (*P* < 0.05). Of the 848 *R. bursa* collected from naturally infested small ruminants and farm dogs, *Babesia ovis* was detected by PCR in salivary glands of 94 adult ticks. The frequency of *B. ovis* infection was higher in flocks with tick in comparison with animals without tick (*P* < 0.05). Positive amplification from blood of ruminants, ticks, oviposition ticks, eggs, and larvae was subjected to restriction digestion with *Hph*I. One RFLP profile was produced. The PCR-RFLP results indicated that one strain of *B. ovis* exists in this area. The results showed that the PCR was useful method to investigate the epidemiology of small ruminants' babesiosis. Furthermore, *R. Bursa*, which can transovarially transmit *B. ovis* and as well as being widely distributed in West Azerbaijan province, Iran, might play an important role in the field as a natural vector of *B. ovis*.

## 1. Introduction

Babesiosis, caused by* Babesia ovis*, is one of the most important tick-borne diseases of sheep and goats in Northwest of Iran and is characterized by apathy, fever, anemia, jaundice, and haemoglobinuria and in some cases mortality may occur.

Knowledge about host-parasite interrelationship has been gainedthrough experimental studies with* B. ovis* [1, 2]; however little is known about the epidemiology and enzootic potential of this parasite.* Rhipicephalus* spp. including* Rhipicephalus bursa*,* R. sanguineus,* and* R. turanicus* have been implicated in the transmission of* B. ovis*, but* R. bursa *has been reported as the only vector for* B. ovis *that can transovarially transmit the* Babesia *parasite to small ruminants. The ticks are widely distributed in most mountainous area of Iran [3]. Since it potentially plays an important role in the transmission of small ruminants babesiosis, it is necessary to investigate the presence status of* Babesia* parasites in this tick.

Diagnosis of babesiosis can be achieved by microscopic examination of Giemsa-stained blood smears and clinical signs in acute phase of the disease, but, after acute infections, recovered animals frequently sustain subclinical infections, which are microscopically undetectable. They can be served as a source of infection for the potential biological vectors causing natural transmission of the disease [4]. Several serological methods standardized for diagnosis of babesiosis have been extensively used in epidemiological studies, but mentioned methods are not specific for any* Babesia* spp. due to the occurrence the cross-reactions with other* Babesia* spp. and the lack of discrimination between acute infection and carrier state. Furthermore, false positive and negative results are commonly observed in these tests [5]. The use of alternative techniques, such as polymerase chain reaction (PCR), has become necessary to detect and identify* Babesia* infections effectively and has been reported in numerous recent studies [6, 7]. Molecular techniques are more sensitive and specific than other traditional diagnostic methods.

Although many analyses were previously performed with the ticks' salivary gland smears stained with methyl-green-pyronin or Feulgen staining, the transmitter agent remained unanswered [8]. Staining of the ticks' salivary glands can definitely confirm the* Babesia* spp. infection of the ticks, but the main drawbacks for this method are the low sensitivity, time-consuming, and the difficulty of differentiating the species involved [9]. Therefore, the application of PCR-based technologies in the epidemiological survey of babesiosis has been reported and high sensitivity and specificity have been verified by several authors for the detection of* Babesia *spp. infection in ticks [10, 11].

Studies on small ruminant's babesiosis in Iran are very limited. Previous serological survey of* B. ovis* performed in different geographical region of Iran [12]. Taking into account the limitation of serological studies, the objectives of the present study was to determine the infection rate in North-West of Iran by PCR. PCR results were compared with the examination of thin blood smear. In addition, the presence of the parasite in salivary glands of* R. bursa *collected from naturally infected sheep, goats, and farm dogs in the region by PCR was performed and PCR-RFLP was employed for identification of* B. ovis* species.

## 2. Materials and Methods

### 2.1. Blood Samples and Ticks Collection

From June 2009 to September 2009, blood samples and ticks were collected from different localities of West Azerbaijan province located in Northwest of Iran. Four hundred two blood samples were collected from 280 sheep and 122 goats that belonged to 40 randomly selected flocks. Flocks were divided into three categories according to their composition: sheep flocks, goat's flocks, and mixed flocks (sheep and goats). Jugular blood samples were collected into tubes containing EDTA for DNA extraction and blood samples from ear vein were obtained for the preparation of thin blood smears, stained with Giemsa and used for the detection of parasites.

During sampling, the whole body of each animal was inspected for the presence of ticks' infestation by palpation. The ticks were manually removed, kept alive in glass tubes, labeled with collection points noted, and then transferred to the laboratory. Two hundred ninety-nine, 213 and 335 adult ticks, respectively, collected from the bodies of sheep, goats, and farm dogs were identified as* R. bursa*; 504 male and 344 female ticks were separated. After determination of parasite burden in some fully engorged female ticks, twenty of them were individually placed on hollow glass plates and incubated at 27 ± 2°C with 75–80% relative humidity for oviposition. On the fifteenth day of oviposition, some of eggs laid were subjected to DNA extraction while others were incubated for hatching into larvae. Finally, the eggs, larvae, and oviposition tick samples were immersed in 70% ethanol and frozen at −80°C for further use.

### 2.2. DNA Extraction and PCR Reaction

Total DNA was extracted from each sheep and goats blood samples using a Genomic DNA purification kit (Fermentas, Germany) and adjusted to a total volume of 200 *μ*L in TE buffer and stored at −20°C until use. Ticks and eggs were rinsed with 70% ethanol and air-dried on the sterile filter paper; DNA was then extracted from each tick and eggs according to the procedure described by Oliviera-Sequeira et al. [11] with some modifications. Briefly, the removed salivary gland was homogenized in 400 *μ*L of homogenizing buffer (0.4 M NaCl, 10 mM Tris-HCl, 2 mM EDTA, pH 8) and then mixed with sodium dodecyl sulphate (SDS) (2% final concentration) and proteinase K (400 *μ*g/mL final concentration). The resultant mixture was incubated at 56°C for 2 h, after which 300 *μ*L of 6 M NaCl was added to the sample. The sample was vortexed for 30 sec and centrifuged at 12000 ×g. The supernatant was transferred to a new tube and an equal volume of isopropanol was added to each sample, mixed well, and samples were incubated at 20°C for 1 h. Samples were then centrifuged for 20 min at 10000 ×g. The pellet was washed with 70% ethanol, dried, and finally resuspended in 50–100 *μ*L sterile dH_2_O.

For DNA extraction from egg samples, 20 mg samples of eggs were weighted out and placed in a microtube and washed with buffer (10 mM Tris-HCL, 1 mM EDTA, 5% Triton X-100, pH 8.5). After centrifugation at 5000 ×g for 2 min, the supernatant was discarded and the eggs were macerated with aid of a glass rod. A solution of Proteinase K (20 *μ*L) was added to the egg macerate and the preparation was incubated overnight at 56°C. The quality of the DNA extract in regard to purity and integrity was assessed with optical density counts at 260/280 nm and submerged gel electrophoresis.

Amplification of thesmall subunit ribosomal RNA (*ssu rRNA*) gene of* Babesia ovis* was performed by sensitive and species-specific primers previously reported and used to amplify a fragment of 549 bp [9]. PCR was carried out in 50 *μ*L total reaction volume containing 5 *μ*L of 10x PCR buffer, 2 mM MgCl_2_, 250 *μ*M of each of the four deoxynucleotide triphosphate, 1.25 U Taq DNA polymerase (Fermentas, Germany), 50 pmol of each primer, and 50 ng of extracted DNA. The sequences of primers were as follows: Bbo-F 5′-TGGGCAGGACCTTGGTTGTTCT-3′, Bbo-R 5′-CCGCGTAGCGCCGGCTAAATA-3′. The positive control for* Babesia ovis* was provided by Professor Rahbari (Faculty of Veterinary Medicine, University of Tehran, Iran). Sterile water served as negative control.

### 2.3. RFLP of PCR Products

The PCR-amplified products were digested with* Hph*I restriction enzyme (Fermentas, Germany) as described by the supplier recommendations. Each digestion reaction was set up in 20 *μ*L volume containing 2 *μ*L of the 10x reaction buffer, 10 *μ*L of PCR products, and 10 units of restriction enzyme. The digestion mixture was incubated at 37°C for 16 h. As control, 20 *μ*L PCR products were treated with 2 *μ*L of the 10x reaction buffer and 8 *μ*L of sterile dH_2_O without adding enzyme. Digested PCR products (expected three fragments 282, 164, and 103 bp) were analyzed at 80 V on 2% agarose gel and visualized under ultraviolet light.

### 2.4. Statistical Analysis

Statistical analysis was performed using the SPSS Software version 17.0 and two-tailed *t*-test. A *P* value less than 0.05 (*P* < 0.05) was considered significant.

## 3. Results

Microscopic examination of thin blood smears showed parasitemia in infected animals ranging from 0.01 to 3% piroplasms, detected inside the red blood cells, pyriform, and single ring. All of these forms were classified as* Babesia *spp. Microscopically, examined blood smears of 280 sheep and 122 goats, 34 (12.1%) and 8 (6.5%), were positive for piroplasms, respectively.

**Figure 1:**
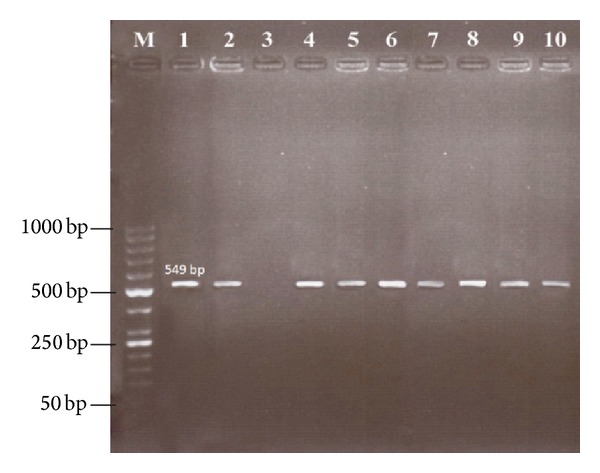
PCR products amplified using* B. ovis*-specific primers. Lane M: 50 bp DNA ladder (Fermentas, Germany), lane 1: infected sheep blood, lane 2: infected goats blood, lane 3: negative control, lane 4: positive control, lane 5: tick collected from sheep, lane 6: tick collected from goat, lane 7: tick collected from dogs, lane 8: tick sample after oviposition, lane 9: egg sample, and lane 10: larvae sample.

Of the 402 examined blood samples, 67 animals (16.7%) yielded a specific* Babesia ovis* ssu rRNA fragment (Figure 1) of which 52 animals (18.5%) were sheep and 15 (12.2%) were goats. All positive samples of sheep and goats by microscopic examination were also positive by PCR. Among 40 examined flocks,* B. ovis* infection was detected in twenty-nine (72.5%) flocks. The percentage of positive animals in each location varied from 13% to 20%. The difference between the prevalence of* B. ovis* infection in sheep and goats was statistically significant (*P* < 0.05).

Tick infestation was determined in all of the animal flocks. A specific fragment of ssu rRNA gene of* B. ovis* was also amplified in 94 out of 848 (11.1%) ticks.* B. ovis* was proved in 12.7% (38/299), 13.6% (29/213), and 8% (27/336) of ticks that were collected from sheep, goats, and farm dogs, respectively. The prevalence of* B. ovis* infection in small ruminants and salivary glands of* R. bursa* are presented in Table 1.

Twenty engorged female ticks were incubated and DNA was extracted from eggs, larvae, and oviposition ticks. Eight oviposition ticks were positive for the specific 549 bp fragment in accordance with its eggs and larvae (Figure 1).

**Figure 2:**
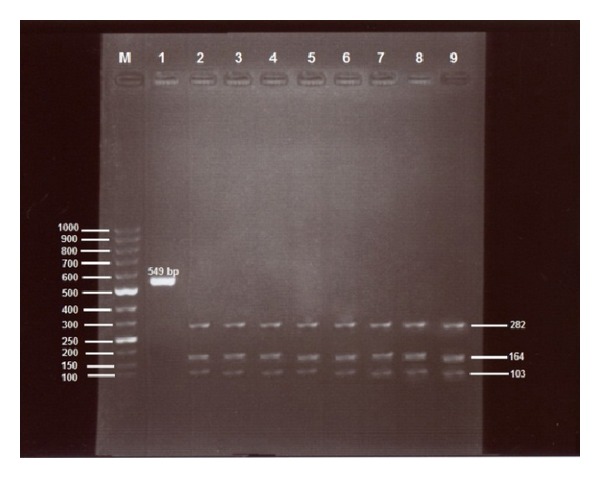
PCR and RFLP profile of amplified 549 bp fragment of the* B. ovis*-specific ssu rRNA gene. Lane M: 50 bp DNA ladder (Fermentas, Germany), lane 1: undigested PCR product, lane 2: digested PCR product from infected sheep, lane 3: digested PCR products from infected goats, lane 4: tick collected from sheep, lane 5: tick collected from goat, lane 6: tick collected from dogs, lane 7: tick sample after oviposition, lane 8: egg sample, and lane 9: larvae sample.

A single RFLP profile was yielded from PCR positive blood samples, ticks, oviposition ticks, eggs, and larvae using* Hph*I restriction enzyme (Figure 2).

## 4. Discussion

The occurrence of* B. ovis*, major causative agent of small babesiosis, had been previously reported in Iran [12–14], but specific prevalence of* Babesia* spp. has been unknown. It has been reported that in the Mediterranean zone [15], Israel [16], Greece [17], Turkey [18], Spain [19], and Iran [20], all showed that* B. ovis* is the most common species affecting small ruminants. The diagnosis of piroplasm infections in vertebrate hosts was mainly carried out by microscopic examination of thin blood smears. However, the method requires expertise because these parasites have similar morphological features and, therefore, may confuse the examiner when mixed infections occur. Serological tests were also used, but there are some difficulties with specificity and sensitivity [5]. An exact differentiation between haemoparasites is crucial to understanding their epidemiology. Molecular methods such as PCR, with a high degree of sensitivity and specificity, have been developed to identify* Babesia* species DNA in affected animals even with 0.00001% parasitemia and their vectors [9, 11, 21, 22]. Identification and characterization of the infected blood and tick samples was performed using pair primers derived from hypervariable area V4 of the small subunit* rRNA* tick sample gene (*18S rRNA*) of piroplasms. The* 18S rRNA* gene has been reliably applied to identify and classify of* Babesia* spp. infection [23].

To our knowledge, the present study is the first molecular diagnostic technique that was employed to determine the molecular epidemiology and infection rate of* B. ovis* in sheep, goats, and vector ticks in Iran. Molecular techniques such as PCR have higher efficiency than microscopic examination and serological assays for detection of* B. ovis* [7, 9].

In the microscopic examination it was found that parasitemia ranges from 0.01 to 3%, while in another study high parasitemia in sheep was reported by [8] and in a study by [17] it was reported that parasitemia never exceeded 1%.

In previous studies in Iran, serological tests employing IFAT was used and the seropositive animals varied from 12% to 58.8% in different regions of the country [12]. In the present study, covering West Azerbaijan province in North-West Iran, the prevalence ranged from 13 to 20% on the farms that were examined.

Among the factor examined in the present study, the species of animals (sheep or goats) and presence of ticks in sheep, goats, and farm dogs were associated with PCR positive results, which indicate a high risk of infection with* B. ovis *in sheep and goats. It is stated that in the field,* B. ovis* causes disease exclusively in sheep, rarely in goats [17, 24]. Infection frequency was significantly higher in ticks collected from the flocks infected with* B. ovis* (27.5%) and was also significantly higher in ticks collected from the sheep infected with* B. ovis* (15%). Our findings indicate the presence of positive relationship between the prevalence of the disease and the presence of vector ticks. The results are in accordance with the findings of previous studies that reported that the frequency of* B. ovis* infection was higher in flocks with tick burden than those in flocks with no tick burden [25, 26]. Frequency of* B. ovis* was higher in ticks collected from goats in comparison with ticks collected from sheep. It was unexpected in this study, because it was observed that* B. ovis* infection frequency was significantly higher in sheep than goats. On the other hand, it is stated that* B. ovis* usually causes disease in sheep, rarely in goats in natural condition [17]. Thus, the reason for the higher* B. ovis* infection in goats may be related to the previous host of the vector tick. It is likely that the ticks transferring from sheep to goats.

Among ticks,* B. ovis* was detected in* Rhipicephalus *spp. from naturally infested small ruminants using PCR application [20]. The finding of* B. ovis* DNA in the salivary glands of ticks is important due to biological transmission of* B. ovis* by* Rhipicephalus* spp. and also it will reveal that the ticks with the* Babesia* DNA in their salivary glands can be considered as* Babesia's* natural transmitter [27].* B. ovis* is transstadially transmitted by* R. sanguineus *[28]. Our results suggest that* R. bursa* may play an important rolein the field as a natural vectorof the parasite that transmit* B. ovis* transovarially.

Previously, RFLP-based assays were used for the detection of bovine babesiosis [29]. In a study on 24 and 35* B. orientalis *infected buffalo blood samples and* R. haemaphysaloides* ticks, respectively, only one RFLP pattern was observed. In our study, 18S rRNA gene yielded just one RFLP profile in 161 PCR products which is in agreement with previous study [29].

In conclusion, the PCR-RFLP assay based on ssu rRNA gene was established successfully and used to investigate the epidemiology of small ruminants' babesiosis in Iran. The results showed that small ruminants' babesiosisexists widely in West Azerbaijan province of Iran and* R. bursa *may be playing an important role in the transmission of* B. ovis* as a natural vector. Sequencing of PCR-amplified products of ssu rRNA gene will clarify more detailed information about genetic diversity of this gene in* B. ovis*.

## Figures and Tables

**Table 1:** Association between the presence of *B. ovis* infection in small ruminants and ticks (*R. bursa*) and the studied parameters (species of animals, origin of ticks).

	Species of animal			Origin of ticks											
	Sheep	Goats	Total	Collected from sheep			Collected from goats			Collected from infected flocks with *B.ovis *	Collected from noninfected flocks	Total	Collected from dogs of infected-flocks with *B.ovis *	Collected from dogs noninfected flocks	Total
				Infected sheep with *B.ovis *	Noninfected sheep	Total	Infected goats with *B.ovis *	Noninfected goats	Total						
Number	280	122	402	93	206	299	75	138	213	215	297	512	198	138	336
Negative	228 (81.4%)	107 (87.8%)	335	79 (85%)	182 (88.4%)	261 (87.3%)	64 (85.31%)	120 (87%)	184 (86.4%)	156 (72.5%)	289 (97.3%)	445 (87%)	176 (88.8%)	133 (96.4%)	306 (92%)
Positive	52 (18.6%)	15 (12.2%)	67	14 (15%)	24 (11.6%)	38 (12.7%)	11 (14.7%)	18 (13%)	29 (13.6%)	59 (27.5%)	8 (2.7%)	67 (13%)	22 (11.2%)	5 (3.6%)	27 (8%)

*P*(*F*)	*P*(*F*) = 0.005			*P*(*F*) = 0.0003			*P*(*F*) = 0.04			*P*(*F*) = 0.003			*P*(*F*) = 0.001		
